# Magnetic Resonance Imaging of the Cervical Spine: Frequency of Abnormal Findings with Relation to Age

**DOI:** 10.3390/medicines8120077

**Published:** 2021-12-14

**Authors:** Ali Alghamdi, Abeer Alqahtani

**Affiliations:** 1 Department of Radiological Sciences, Faculty of Applied Medical Sciences, University of Tabuk, Tabuk 71491, Saudi Arabia; 2 Department of Radiology, King Fahad Hospital, Albaha 65515, Saudi Arabia; abeerzumaya@hotmail.com

**Keywords:** magnetic resonance imaging, cervical spine, age-related, findings

## Abstract

Background: Patients with neck pain are frequently encountered in cervical spine (C-spine) magnetic resonance imaging (MRI) practice. However, the exact distribution and prevalence of cervical abnormalities are not known. Aim: The aim of this study is to evaluate the association between age, gender, and prevalence of abnormal cervical MRI findings. Methods: Records of 111 cervical MRIs were collected in 12 months from January to December 2019 from adults aged 20–89 years who were referred from neurosurgery, neurology, and orthopedic clinics. Findings were classified and analyzed using the Statistical Package for Social Science (SPSS), version 24.0 (IBM, Armonk, NY, USA). The chi-square test was used to determine the association between demographics and abnormalities using a significance of *p* = 0.05. Results: The majority of patients were female (72.1%). The number of abnormal incidences increased with age until it reached a peak at ages 50–59. Spondylodegenerative changes were the most frequent finding, which was present in 52.2% of the total sample, and was followed by disc bulge (25.2%). Incidences increased in lower discs, with C5–C6 being the most frequent in 65% of the total sample. Younger males in their 20s had more injuries than females of the same age. However, this rate was reversed in patients over 40, as women were the dominant gender among patients in their 40s with cervical injuries, with a rate of 81.5%. Conclusion: In our study, we found that older patients developed more C-spine injuries. Gender may play a role in the rate of incidents. However, we did not find any significant differences between men and women or between different types of abnormalities.

## 1. Introduction

Magnetic resonance imaging (MRI) is a noninvasive imaging technique that is usually used to investigate the potential causes of neck pain. Differences in MRI findings among people are expected to be associated with age, gender, and quality of life.

In the recent decades, there has been an increasing rate and cost of cervical spine surgeries [1]. A wide range of research about the epidemiology, management, morbidity and mortality of abnormal findings in the cervical spine has been added to the literature in the last few years [2,3,4,5,6,7,8]. Degenerative diseases in the cervical spine (C-spine) are the most common neurological disorders in the older population. The progress of these diseases may lead to different injuries, such as cervical spondylosis and disc herniation. The prevalence of degeneration in the spine in the geriatric population requires a comprehensive understanding of clinical history and physical examinations, which are beyond the scope of this study. Imaging findings can therefore be associated with these outcomes.

The relationship between degenerative changes seen in C-spine MRI and neck symptoms is still unclear [2,3,4,5,6,7,8]. It has been proven that degenerative findings in the C-spine in asymptomatic subjects are age dependent [6,9,10,11,12]. The number of patients with cervical spondylotic myelopathy has been shown to increase with age [9]. Incidences of spinal stenosis also increase in elderly patients [9]. Degenerative diseases sometimes present as normal aging variations [10].

Although plain radiography provides valuable information about the C-spine, advanced imaging, such as computed tomography (CT) and MRI, plays a crucial role in providing an accurate diagnosis. However, to avoid the high radiation dose of CT, MRI is a great choice. Several previous studies using plain radiography have established the normal morphology and kinematic behavior of the C-spine [3,4,5,6].

No studies in Saudi Arabia have evaluated the influence of age on MRI C-spine findings for either asymptomatic or symptomatic patients. The definition, classification, and association of C-spine injuries and diseases are of specific interest for providing comprehensive understanding regarding the risk factors of the most common injuries and diseases, especially regarding those preoperative cases. The purpose of this study is to investigate the association between age and abnormal findings in C-spine MRIs in nontraumatic adult patients who suffer neck pain.

## 2. Methods

### 2.1. Patients

This is a retrospective cross-sectional study. A total of 111 records of cervical MRIs for adult patients aged 20–89 years with neck pain were reviewed using a 1.5 T superconductive magnet for 12 months from 1 January 2019 to 30 December 2019. Patients commonly were referred from neurology, neurosurgery, or orthopedic clinics in King Fahad Hospital, which serves as the major public hospital in Al-Baha, Saudi Arabia. The majority of patients lived within the city limits of Al-Baha. The exclusion criteria included a history of coronal deformity, trauma, brain or spinal surgery, and MRI images with artefacts. Inclusion criteria included all outpatients with neck pain, and sensory or motor symptoms, such as numbness, motor weakness, clumsiness, or gait disturbance.

Age groups were classified based on decades (e.g., 20–29). Abnormal findings were classified according to different categories. For example, curvature status was divided into normal and straightened and the six locations of cervical abnormality in the vertebral discs were classified from cervical 1–cervical 2 (C1–C2) to C7–T1.

### 2.2. Ethical Approval

Ethical approval was obtained from the institutional review board of the Scientific and Research Committee at King Fahad Hospital, Al-Baha (approval number KFHBAHA/17022021/7). MRI reports were analyzed by an expert consultant radiologist.

### 2.3. Statistical Analysis

The data were analyzed using Statistical Package for Social Science (SPSS, version 26, IBM Corp, Armonk, NY, USA). The chi-square test was used to evaluate differences in abnormal findings between different groups. We analyzed the association between findings and demographic data and how different findings are linked to each other—for example, how frequently disc bulge is associated with spinal cord compression and how much spinal cord compression would increase signal intensity ISI incidence. Statistical significance is considered as *p* < 0.05.

## 3. Results

This study reviewed records of 111 patients who were referred to MRI for C-spine assessment. Of the total patients, males comprised 27.9% and females comprised 72.1%. The distribution of patients according to age is shown in Table 1.

The number of cervical MRI cases increased gradually with age until it reached the most populated group at ages 50–59 (*n* = 41, 36.9%), followed by the age group 40–49 (*n* = 27, 24.3%), and then it dropped gradually until it reached the least number of patients at ages 80–89 (*n* = 3, 2%). Of the total patients, 54.1% reported a straightened C-spine. Our results show that a straightened C-spine was observed in about 55% of female patients and 52% of male patients.

**Figure 1:**
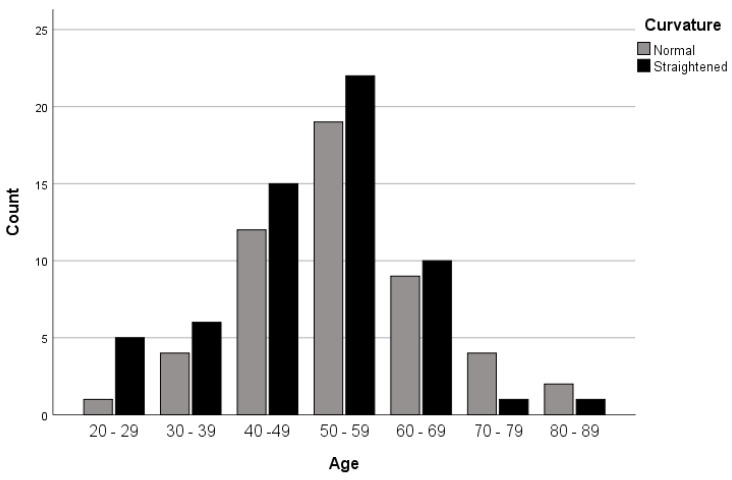
Prevalence of normal and straightened C-spine in different age groups.

An abnormal (straightened) curvature increased with age gradually from the 20s until it reached its peak at the 50s (Figure 1). That is to say, among the overall patients with a straightened C-spine, there were only 8.3% aged 20–29 and 36.7% aged 50–59.

The most frequent finding was spondylodegenerative changes (Table 2), which were present in 52.2% of patients, followed by disc protrusions and a disc bulge in 25.2% and 11.7% of patients, respectively. The least frequent abnormality was a burst fracture in 1.8% of cases.

**Figure 2:**
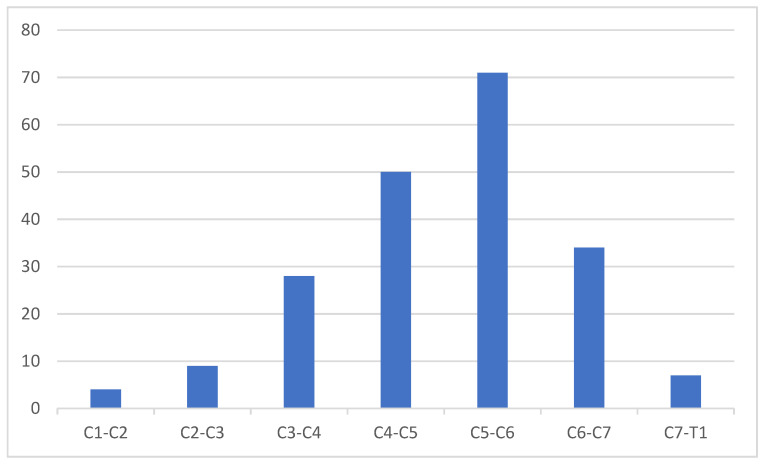
Most frequently injured cervical discs.

Figure 2 shows the frequency of evidence according to vertebral discs in the C-spine. Our findings show that abnormalities increase gradually in lower discs until they reach the higher rates of 64% of patients with injuries in C5–C6. Then, the number of abnormalities drop gradually toward C6–C7.

**Figure 3:**
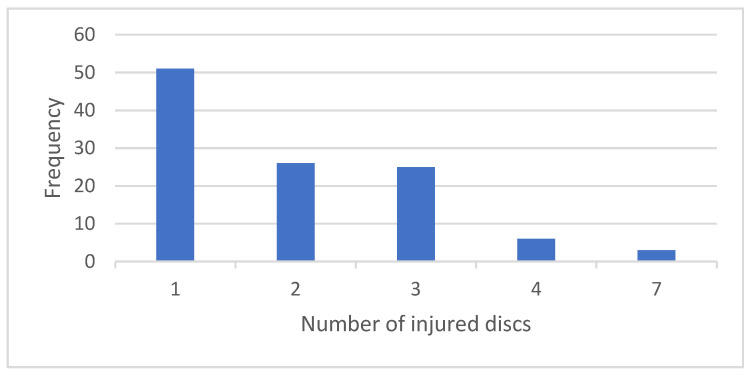
Number of injured discs in patients.

Figure 3 shows the frequency of cases according to the number of injured discs. The reviewed records show that most patients (46%) had abnormalities in only one disc. Only three patients had injuries in all seven cervical discs.

The type of injury was significantly associated with age (*p* = 0.001). That is to say, 44.8% of patients with spondylodegenerative changes were aged 50–59, and only 3.4% of patients aged 20–29 developed spondylodegenerative changes. Patients in the 40–49 age group comprised the highest rate of all patients with disc protrusions (43%), followed by those aged 50–59 (21.4%).

**Figure 4:**
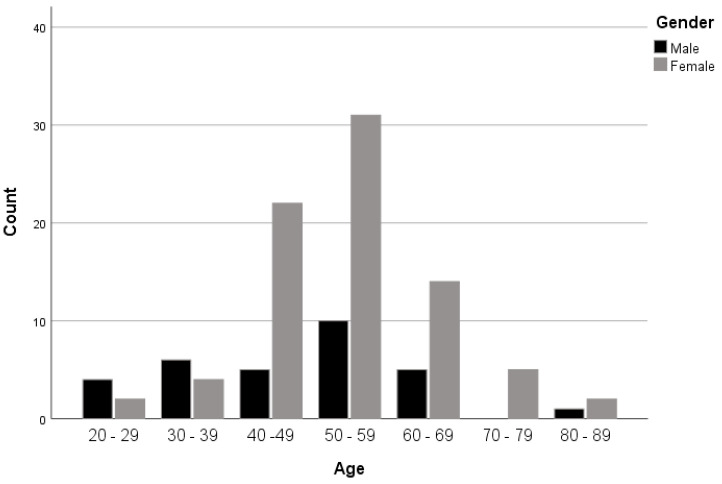
Distribution of gender in each age group.

Surprisingly, abnormalities in different age groups were gender dependent (*p* = 0.043). In the younger groups, more male patients were observed (Figure 4). For example, 66.7% of patients in the 20–29 age group were males and only 33.3% were females. In addition, 60% of the 30–39 age group were males, while 40% were females. However, this rate was reversed in patients over 40. That is to say, 81.5% of patients in the 40–49 group were females, while only 18.5% were males; females comprised 75.6% of patients in the 50–59 age group and only 24.4% were males; and females maintained higher rates compared to males in all older groups.

## 4. Discussion

Understanding the association between age and the number of incidents of an abnormal C-spine can help more effective medical [11], surgical [12], and rehabilitative management [13]. To our knowledge, few studies have evaluated MRI findings of the C-spine in relation to age. Evidence presented in our study indicates that, over a period of 12 months (between 1 January 2019 and 30 December 2019), abnormal MRI findings in the C-spine were significantly related to age.

Although abnormal C-spine curvature increased with age, no significant difference was observed between different age groups. In addition, no significant differences between male and female patients were observed. Our study showed that a majority of abnormal findings were found in female patients. Further study is required to investigate the factors influencing the incident rate in our study. This is consistent with previous studies that showed higher rates in female patients in the presence of different factors. For example, neck pain in female patients was significantly higher than that of male patients in the preceding 12 months of the radiographic examination. In addition, there was an association between degenerative changes and severity of neck pain in female patients [14]. This was supported by another study in which women more frequently exhibited thoracic (TX) and cervicothoracic junction (CTJ) pain with a wider lateral spread, regardless of disease status [15].

This was consistent with a previous study [16]. However, in that study, the authors found a significant difference in curvature profile between those over and under 40 years old. The authors suggested this may be due to age-related back muscle strength. One reason could be that, in the supine position, the physiological curvature becomes smaller than it is in the erect position [16]. An increase in cervical lordosis in both men and women has also been reported [17]. By contrast, Gote et al. found that cervical lordosis differed with age in men and women [18], as it appears earlier in men than in women.

**Figure 5:**
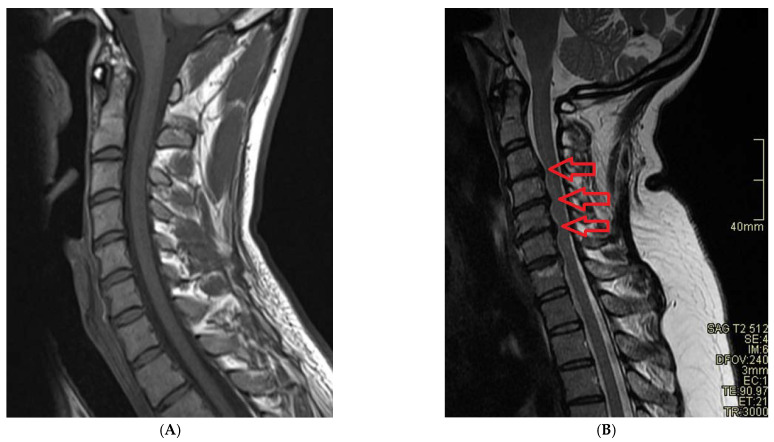
(**A**) Normal sagittal cervical spine T1 MRI; (**B**) 50 year old female patient with straightened cervical curvature is noted due to muscle spasm, in addition to advanced spondylodegenerative changes and multiple disc bulges (red arrows) of the cervical vertebrae. Reduced hydration of all cervical discs and sub end plate marrow degenerative changes in C3–C4, C4–C5 are also observed.

In addition, although we found fewer abnormal findings in the higher vertebral levels, our findings show that vertebral disc abnormalities in the C-spine were more frequent in the lower discs, with the most injured disc being C5–C6 (Figure 2). This could be due to increased pressure caused by weight on the lower discs. In relation to age, no disc bulging was observed in younger adults in their 20s. However, the rate of disc bulging (Figure 5) increased with age from the 20s to a peak at the 50s, as follows: 0% of the 20–29 age group had a disc bulge, 7.7% among ages 30–39, 23.1% among ages 40–49, a peak of 38.5% among 50–59, and then a drop in the older groups until it reached 0% among ages 80–89 (*p* > 0.05). The reason for the decreased cases of bulging disk with increased age could be due to the fact that a part of disc bulging cases resulted from physical injuries such as sport and motor vehicle injuries, which are increasing in younger ages worldwide [19]. Therefore, disc bulging decreases as the incidence of traumatic injuries decreases, which is the case in older population.

The increase of incidents of cervical disc bulging with age has been reported previously in asymptomatic patients [9,20,21,22,23,24]. The number of cases increased and reached a peak for the 50s age group. Disc bulging and protrusions occurred more frequently in lower levels.

It has been reported that age-related progressive changes in the intervertebral discs begin in the third decade of life [13]. Age-related physiological and pathological changes in the intervertebral discs have been outlined previously by Coventry et al. [25,26]. In some case, the intervertebral disc alterations are recognizable even to the naked eye [27]. However, MRI may be needed to detect signs of degeneration in asymptomatic persons [28,29].

**Figure 6:**
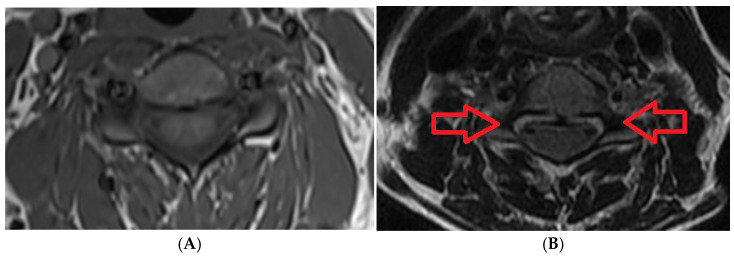
(**A**) Normal axial T1 MRI of cervical spine; (**B**) 57 year old female patient with C3–C4, C4–C5, C5–C6 and C6–C7 abnormalities: multiple levels of posterior disc osteophytic complexes indenting the ventral aspect of the subarachnoid space and encroaching upon neural foramina bilaterally at the corresponding levels.

Moreover, our results show that degenerative changes (Figure 6) in the C-spine were significantly age dependent (*p* < 0.05). For example, of the total patients with spondylodegenerative changes, 44.8% were in their sixth decade, and only 3.4% were in their third decade. This was consistent with a previous study conducted by Friedenberg and Miller [30], in which two large groups, one symptomatic and the other asymptomatic, were examined using x-rays of the spine. The authors observed degenerative changes in 75% of patients in their seventh decade and 25% of patients in their fifth decade. In both groups, the abnormalities were most frequent at the C6–C7 levels. The incidences of disc space narrowed at C5–C6 and were higher at C6–C7 in symptomatic patients [30]. A recent study reported that the age dependent degenerative changes of the cervical spine were more notable in females [31]. The effects of aging on the profile and alignment of the cervical spine have been recently reported [32,33].

There are several limitations to our study. First, it is a single-site study with a small sample size. Thus, a comprehensive study that includes several sites with a greater population is required to generate fuller results. Second, no information about quality of life or daily practices was collected. These factors may be associated with the degree of abnormal findings. In addition, MRI images were taken in the supine position, which is not a natural position of the spine. Therefore, X-rays could provide valuable information. Moreover, no follow-up was taken to assess the additional changes that may develop with aging. Therefore, a cohort study would provide valuable information about age-related changes.

## 5. Conclusions

In our study, we have shown that younger male patients (in their 20s) are more prone to C-spine diseases than females of the same age, while older women (over 40) are more affected by the same diseases than men of the same age. Lower cervical discs are more frequently affected by disc diseases than higher vertebral discs. Abnormal findings also increase with age. However, we did not find any significant differences between men and women or between different types of abnormalities. This study provides valuable information about the distribution of abnormal findings of cervical spine MRI. This would be useful for MRI units and neurology clinics to set the best management of referral pattern, reduce waiting time and increase cost efficiency.

## Figures and Tables

**Table 1:** Distribution of the sample according to gender.

			Gender		Total
			Male	Female	
Age	20–29	Count	4	2	6
		% within age	66.7%	33.3%	100.0%
	30–39	Count	6	4	10
		% within age	60.0%	40.0%	100.0%
	40–49	Count	5	22	27
		% within age	18.5%	81.5%	100.0%
	50–59	Count	10	31	41
		% within age	24.4%	75.6%	100.0%
	60–69	Count	5	14	19
		% within age	26.3%	73.7%	100.0%
	70–79	Count	0	5	5
		% within age	0.0%	100.0%	100.0%
	80–89	Count	1	2	3
		% within age	33.3%	66.7%	100.0%
Total		Count	31	80	111
		% within age	27.9%	72.1%	100.0%

**Table 2:** Number of incidences.

	Number	Percentage %
Disc protrusions	28	25.2
Disc bulge	13	11.7
Mild spondylodegenerative changes	58	52.3
Disc osteophytic complexes	10	9
Burst fracture	2	1.8
